# Effect of Peptide–Polymer Host–Guest Electrostatic Interactions on Self-Assembling Peptide Hydrogels Structural and Mechanical Properties and Polymer Diffusivity

**DOI:** 10.1021/acs.biomac.4c00232

**Published:** 2024-05-21

**Authors:** Siyuan Dong, Sam L. Chapman, Alain Pluen, Stephen M. Richardson, Aline F. Miller, Alberto Saiani

**Affiliations:** † Department of Chemical Engineering, School of Engineering, Faculty of Science and Engineering, The University of Manchester, Oxford Road, M13 9PL Manchester, U.K.; ‡ Manchester Institute of Biotechnology (MIB), Faculty of Science and Engineering, The University of Manchester, Oxford Road, M13 9PL Manchester, U.K.; § Division of Pharmacy and Optometry, School of Health Sciences, Faculty of Biology, Medicine and Health, The University of Manchester, Oxford Road, M13 9PL Manchester, U.K.; ∥ Division of Cell Matrix Biology and Regenerative Medicine, School of Biological Sciences, Faculty of Biology, Medicine and Health, Manchester Academic Health Science Centre, The University of Manchester, Manchester M13 9PT, U.K.

## Abstract

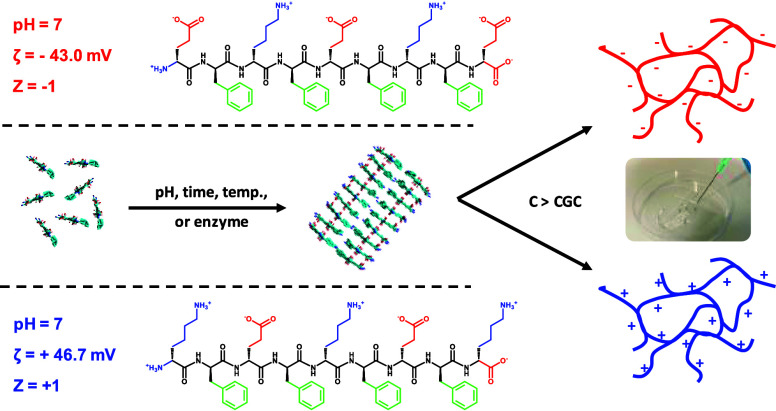

Peptide-based supramolecular hydrogels are an attractive class of soft materials for biomedical applications when biocompatibility is a key requirement as they exploit the physical self-assembly of short self-assembling peptides avoiding the need for chemical cross-linking. Based on the knowledge developed through our previous work, we designed two novel peptides, *E*(FKFE)_2_ and *K*(FEFK)_2_, that form transparent hydrogels at pH 7. We characterized the phase behavior of these peptides and showed the clear link that exists between the charge carried by the peptides and the physical state of the samples. We subsequently demonstrate the cytocompatibility of the hydrogel and its suitability for 3D cell culture using 3T3 fibroblasts and human mesenchymal stem cells. We then loaded the hydrogels with two polymers, poly-l-lysine and dextran. When polymer and peptide fibers carry opposite charges, the size of the elemental fibril formed decreases, while the overall level of fiber aggregation and fiber bundle formation increases. This overall network topology change, and increase in cross-link stability and density, leads to an overall increase in the hydrogel mechanical properties and stability, i.e., resistance to swelling when placed in excess media. Finally, we investigate the diffusion of the polymers out of the hydrogels and show how electrostatic interactions can be used to control the release of large molecules. The work clearly shows how polymers can be used to tailor the properties of peptide hydrogels through guided intermolecular interactions and demonstrates the potential of these new soft hydrogels for use in the biomedical field in particular for delivery or large molecular payloads and cells as well as scaffolds for 3D cell culture.

## Introduction

Hydrogels are a fascinating class of materials that find applications across a variety of fields. Their biphasic nature and low level of structural order make them challenging materials to design and characterize. Supramolecular hydrogels in particular have come to the fore over the last few decades as they allow the building of typically soft hydrogels through the guided self-assembly of small molecules avoiding chemical cross-linking. This is an attractive preparation route for biomedical applications when biocompatibility is a key requirement.^1−3^ In this context, self-assembling peptides are a particularly relevant class of molecular building blocks. Built from nature’s 20 amino acid toolbox, they can be designed to be biocompatible with low immunogenicity; being usually made through standard solid-phase synthesis, they can be produced with high definition and purity, allowing the design of fully defined systems. These properties make them particularly attractive for the design of hydrogel scaffolds for cell culture^4,5^ and tissue engineering^6,7^ or for the design of hydrogel carriers for in vivo delivery of drugs^5^ and cells.^8,9^ The formulation of such materials for the latter application requires not only an in-depth understanding of peptide self-assembly and gelation processes across length scales but also of the effect of incorporating guests, whether cell or drugs, on the self-assembly pathway and the long-term stability of the hydrogels.

A variety of peptide designs can be found in the literature that allow the formulation of stable hydrogels.^10,11^ In our group, we focus on a particular design developed originally by Zhang and co-workers which is based on short peptides, typically 4–20 amino acids long, with alternating hydrophilic (e.g., glutamic acid, lysine, aspartic acid, arginine) and hydrophobic (e.g., phenylalanine, valine, leucine, isoleucine, tyrosine, tryptophan) residues.^12−14^ The key property of this family of peptides is their ability to self-assemble in water-based media into cross β-sheet fibers that above a critical gelation concentration (CGC) entangle and associate to form 3D swollen networks, in other words, hydrogels.^15,16^ These cross β-sheet fibers are thought to derive from the assembly of two antiparallel β-strands through their hydrophobic faces, resulting in the hydrophobic residue side chains being located in the core of the fibers while the hydrophilic residue side chains are located on the surfaces of the fibers. This typically leads to the formation of rectangular twisted elementary fibers with widths of ∼3 to 10 nm depending on the length of the peptide sequence and thicknesses of 1.1–1.3 nm depending on the nature of the hydrophobic residues side groups.^17−20^ These fibers can then either associate and entangle to form networks and hydrogels or further self-assemble into more complex structures, such as tubes, sheets, and ribbons.^21^

In our recent work, we have shown how by design we can modify the fiber core,^22,23^ surfaces,^24,25^ and edges^26^ of a family of phenylalanine-based peptides to control the physicochemical properties of these elementary fibers and consequently tailor the physical and mechanical properties of the hydrogels to the targeted application. In the context of drug delivery, more recently, we have shown how the hydrophilic residues side chains present on the surface of the cross β-sheet fibers can be used to control the release of small molecules, including doxorubicin, through secondary interactions, such as electrostatic, π–π, cation-π, and hydrophilic/hydrophobic.^27−29^

**Figure 1:**
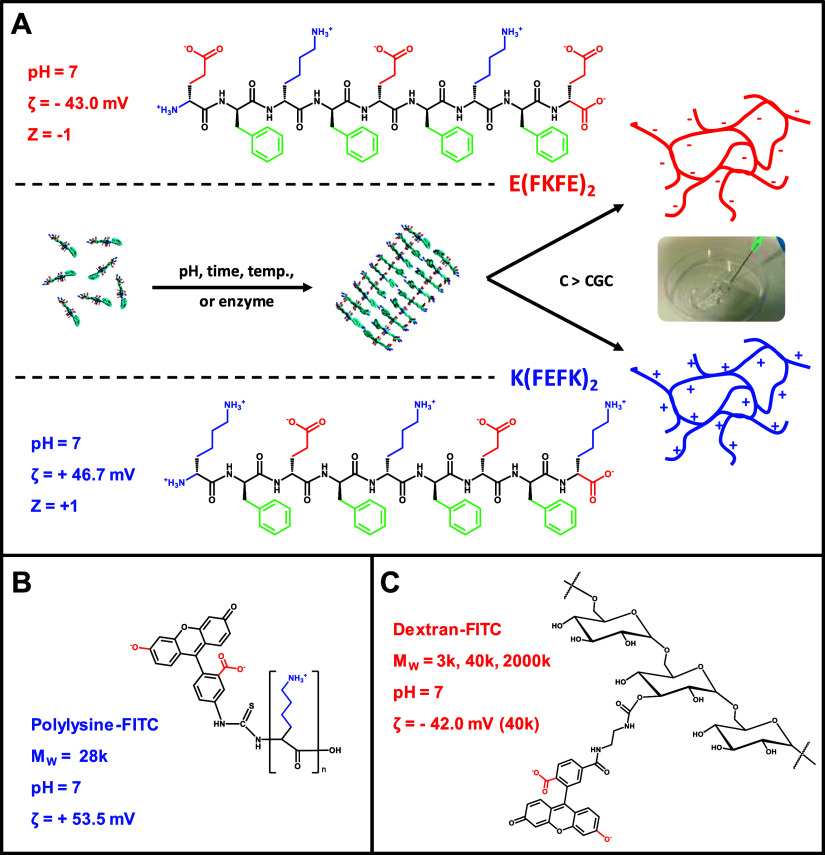
(A) Chemical structures and physicochemical properties at pH 7 (ζ zeta potential and Z theoretical charge) of the two peptides used – Top: *E*(FKFE)_2_, Bottom: *K*(FEFK)_2_, and Middle: schematic representation of the self-assembly pathway of this family of peptides. (B and C) Chemical structures and physicochemical properties at pH 7 (*M*_w_: average molecular weight in g mol^–1^) of poly-l-lysine and dextran used.

In this current work, we were interested in designing novel cytocompatible peptide hydrogels that gel at pH 7 and understand how electrostatic interactions can be exploited to control the release of high molecular weight molecules such as polymers. For the purpose of this specific work, we designed two new “symmetrical” sequences: *K*(FEFK)_2_ and *E*(FKFE)_2_ with K being lysine, E glutamic acid, and F phenylalanine (Figure 1A). Both have hydrophilic terminal residues K and E, respectively, limiting fiber edge hydrophobic interactions and therefore cross-linking.^26^ They will carry between pH 6 and 8 overall opposite theoretical charges with moduli of 1, +1 for *K*(FEFK)_2_ and −1 for *E*(FKFE)_2_, allowing in theory the formulation of stable and transparent hydrogels at physiological pH.^20,25,26,29^

In the first part of this work, we investigate the phase behavior of these peptides in water has a function of pH and concentration. Subsequently, we characterized the hydrogel structures and properties using a range of techniques including attenuated total reflectance Fourier-transform infrared spectroscopy (ATR-FTIR), transmission electron microscopy (TEM), small-angle X-ray scattering (SAXS), and shear rheology. We then investigated the basic cytocompatibility of the hydrogels through 3D cell culture using two cell lines, mouse 3T3 fibroblasts, and human bone marrow-derived mesenchymal stem cells (MSCs). Cell viability and proliferation were assessed using live/dead and PicoGreen assays, respectively.

As high molecular weight guest molecules, we chose two fluorescein (FITC)-labeled polymers carrying opposite charges as pH 7, poly-l-lysine and dextran. Poly-l-lysine (average molecular weight, *M*_w_ ∼ 28,000 g mol^–1^) will carry a positive charge at pH 7 (Figure 1B), while dextran will carry a negative charge (Figure 1C). For the latter polymer, three different average molecular weights were used, *M*_w_ ∼ 3000, ∼ 40,000, and ∼2,000,000 g mol^–1^, to probe the contribution of molecular size to the diffusion of these guest molecules out of the hydrogels. We investigated first the effect that incorporating these polymers into the hydrogels has on their structure, mechanical properties, and swelling behavior and, subsequently, the diffusion of the polymers out of the hydrogels using UV–vis spectroscopy.

## Materials and Methods

### Materials

The peptides used in this study were purchased from Karebay Biochem, Inc., as HCl salts with a nominal sequence purity of 95%. Peptide sequence purities were confirmed by reverse-phase high-performance liquid chromatography. Fluorescein-labeled dextran (*M*_w_: 3, 40, and 2000 kDa) was purchased from Thermo Fisher Scientific, while fluorescein-labeled poly-l-lysine (*M*_w_: 28 kDa) was purchased from Sigma-Aldrich. All polymers were used as received. The physicochemical properties of the polymers used are summarized in Table 1.

**Table 1:** Polymer Physical Properties: Zeta Potential, Hydrodynamic Radius, and Diffusion Coefficients

polymer	zeta potential,^a^ ζ (mV)	hydrodynamic radius,^b^*r*_H_ (nm)	diffusion coefficient,^c^*D*_0_ (m^2^ s^–1^)	charges per monomer^d^
3kDex	–32.9 ± 2.7	1.4 ± 0.1	(1.50 ± 0.09) × 10^–10^	0.027–0.054
40kDex	–42.0 ± 3.8	7.7 ± 0.3	(2.77 ± 0.10) × 10^–11^	0.024–0.008
2000kDex		43.8 ± 2.1	(4.87 ± 0.23) × 10^–12^	0.01–0.001
28kPLys	+53.5 ± 3.4	4.2 ± 0.3	(5.11 ± 0.40) × 10^–11^	1

a Zeta potentials (ζ) were measure at 37 °C and pH 7 using a Zetasizer Nano (Malvern Instruments Ltd., UK). Samples were prepared at 0.2 mg mL^–1^ peptide concentration in HPLC grade water and pH adjusted to 7 using an 0.1 M NaOH solution.

b Diffusion coefficients were measured using an LSM 510 META/Confocor 2 system (Zeiss, Jena, Germany) equipped with an argon laser and a 40× /1.2NA water-immersion objective. 3kDex, 40kDex, and 28kPLys polymer solutions were prepared at 100 nM, while 2000kDex solution was prepared at 10 nM in HPLC grade water.

c Hydrodynamic radii were calculated using the Stokes–Einstein equation, , where *r*_h_ is the hydrodynamic radius, *k*_b_ is the Boltzmann constant, *T* is the temperature in Kelvin, *D*_0_ is the diffusion coefficient, and μ is the viscosity.^30^

d Estimated using supplier information.

### Peptide Titrations

Peptide titration experiments were performed by adding 0.1 or 0.5 M NaOH solutions in 1–2 μL steps to a 1 mL HPLC-grade water peptide solution with a 2 mg mL^–1^ starting concentration. After each NaOH addition, the samples were vigorously agitated using a vortex to ensure homogeneous mixing and the pH was measured using a Fisherbrand Hydrus 300 benchtop pH meter.

### Physical-State Phase Diagrams

Initial samples were prepared by dissolving the required amount of peptide powder in 2 mL of HPLC-grade water in a 5 mL Eppendorf tube. The samples were mixed for 30 s using a vortex before measuring the initial pH and recording the initial physical state. Two microliters of a 0.5 M NaOH solution were then added stepwise. After each addition, the samples were mixed for 30 s, and their pH and physical state were recorded. Samples were deemed solution when they flowed upon inversion of the tube, transparent/cloudy hydrogels gels when they did not flow upon inversion of the tube, and precipitated when clear macroscopic phase separation was observed.

### pH 7 Hydrogel Preparation

Peptide hydrogels at pH 7 were prepared in 5 mL batches by dissolving the required amount of peptide and polymer (for polymer-loaded hydrogels) powders in 3.5 mL of HPLC grade water and mixing for 30 s. The sample pH was then adjusted to 7 by the addition of the required amount of a 0.5 M NaOH solution as established through the titration experiments. The samples were then mixed vigorously and gently centrifuged, if necessary, to remove any bubbles. Their pH was then measured and adjusted if required through further small additions of NaOH. Once pH 7 was achieved, the required HPLC-grade water was added to achieve the target concentration and batch volume. The samples were then mixed once more and stored (at least 12 h) in a fridge before use.

### Attenuated Total Reflectance Fourier-Transform Infrared Spectroscopy

Hydrogel samples were prepared as described earlier. ATR-FTIR measurements were performed on a Bruker VERTEX 80 FTIR spectrometer equipped with a single-bounce diamond ATR accessory. A small drop of hydrogel was placed on the surface of the diamond and pressed in position using a spatula to ensure good contact between the hydrogel and the diamond surface. The beam path was purged with dry CO_2_-scrubbed air. The spectra were an average of 256 scans collected using a 4 cm^–1^ resolution. An HPLC-grade water spectrum was used as a background and subtracted from each sample spectrum.

### Transmission Electron Microscopy

Hydrogels were prepared as described earlier and diluted 20-fold using HPLC-grade water. The samples were vigorously mixed with a vortex to separate as much as possible the fibers. A carbon-coated copper grid (400 mesh grid Electron Microscopy Sciences, Hatfield, Pennsylvania, USA) was glow discharged and charged negatively. The carbon copper grid was then placed sequentially on a 10 μL sample droplet for 60 s, a 10 μL droplet of HPLC grade water for 10 s three times, a 10 μL droplet of 5% uranyl acetate solution for 30 s, and finally, on a 10 μL droplet of HPLC grade water for 10 s. After each step, excess liquid was drained off using lint-free tissue (90 mm Whatman 1). The grid was then left to air-dry for 2–5 min. TEM images were taken using an FEI Tecnai12 BioTwin transmission electron microscope running at 100 kV and equipped with a Gatan Orius SC1000A CCD camera. Fiber width analysis was performed by measuring randomly the widths of 700 fiber (cut-off size 20 nm) using ImageJ software across at least three TEM images for each sample.

### Small-Angle X-ray Scattering

SAXS experiments were performed on beamline I22 at the diamond light source (DLS) synchrotron (Didcot, UK).^31^ The energy of the beam was 12.4 keV, corresponding to an X-ray wavelength of 0.1 nm. Quartz capillaries (1.5 mm outer diameter and 0.01 mm wall thickness) from Capillary Tube Supplies Ltd. (Bodmin, UK) were used as sample holders. The samples were prepared as described earlier and injected into the capillaries using a syringe. The sample-to-detector distance was fixed to 5.77 m, corresponding to an accessible momentum transfer vector range of 0.1 nm^–1^ < *q* = (4π/λ) sin(θ/2) < 3.0 nm^–1^, where θ is the scattering angle and λ is the wavelength of the incident photons. Calibration of the SAXS detector (Pilatus P3–2M, Dectris, Switzerland) was performed using silver behenate powder, and data were collected as 10 × 100 ms frames. Data were reduced using the Dawn software suite available from DLS.^32^ The 2D isotropic scattering patterns were corrected for the detector response, dark current background, and sample transmission and then azimuthally integrated to generate 1D scattering patterns. Under these conditions, the sample coherent normalized scattering intensity *I*_N_(*q*) is
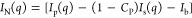
1where *I*_p_(*q*) is the normalized intensity scattered by the sample, *I*_s_(*q*) is the normalized intensity scattered by the solvent in our case HPLC grade water, *C*_p_ is the peptide concentration in g cm^–3^, and *I*_b_ is the background scattering originating mainly from the incoherent scattering of the peptides. *I*_s_(*q*) was obtained by measuring the scattering of the solvent, HPLC grade water, and *I*_b_ was estimated using the Porod law that gives the scattered intensity of a two-phase system at high *q* values:
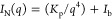
2where *K*_p_ is the Porod constant. *I*_b_ was estimated by fitting the last 40 data points of the 1D scattering patter using a Porod plot [*q*^4^*I*(*q*) vs *q*^4^].^33−36^

### Oscillatory Shear Rheology

Rheological measurements were performed using a Discovery Hybrid 2 (DHR-2) rheometer from TA Instruments (New Castle, Delaware, USA) using a 20 mm parallel plate geometry and a 500 μm gap. Samples were prepared as described earlier. Hydrogels (200 μL) were pipetted onto the rheometer’s static bottom plate and the rheometer top plate lowered to the desired gap size. For hydrogel in cell culture inserts and conditioned with cell culture media (see below), the bottom membranes of the insets were removed, and the hydrogels deposited on the rheometer’s static bottom plate. Samples were covered with a solvent trap to avoid evaporation and left to equilibrate at 37 °C for 180 s before the experiments. Strain sweep experiments were performed at 1 Hz in the 0.01–100% strain range. The shear-thinning and recovery (i.e., injectability) experiments were performed by applying sequentially a low shear strain (0.2%) for 5 min followed by a high shear strain (1000%) for 1 min and again a low shear strain (0.2%) for 5 min.

### Swelling Experiments

Hydrogels were prepared as described earlier at 12 mg mL^–1^, and 1 mL was transferred into 5 mL cylindrical glass vials. Vials were gently tapped on the bench to eliminate any bubbles and obtained flat surfaces, and the height of the hydrogels was measured (day 0). Three milliliters of HPLC-grade water was then added on the top of the hydrogels. After being stored the samples for 10 days at 37 °C, the vials were inverted, and the height of the hydrogels was measured once again (day 10). The swelling ratio (*Q*) was defined as (*H*_1_ – *H*_0_)/*H*_0_, where *H*_0_ is the height of the hydrogel at day 0 and H_1_ is the height of the hydrogel at day 10.

### Polymer Release Experiments

To establish the standard UV absorbance curves, polymer solutions were prepared by dilution at 1, 0.8, 0.6, 0.4, and 0.2 mg mL^–1^. UV absorbance was measured using a Jenway 6715 UV/vis spectrophotometer at 505 nm. Polymer-loaded hydrogels were prepared as described earlier at concentrations of 12 mg mL^–1^ for peptides and 0.8 mg mL^–1^ for polymers, and 1 mL of polymer-loaded hydrogel was transferred into 5 mL cylindrical glass vials. Vials were gently tapped on the bench to eliminate any bubbles and obtain flat surfaces. Three milliliters of HPLC grade water was then added on the top of the hydrogels, and the samples were stored at 37 °C. At each time point (0.25, 0.75, 1.25, 2, 4, 12 h and thereafter every 12 h up to 120 h, i.e., 5 days) 1 mL of supernatant was collected, its UV absorbance at 505 nm measured and then placed back in the glass vial on the top of the sample. Polymer concentrations in the supernatant were then calculated using the standard curves.

### 3D Cell Culture

3T3 mouse fibroblast and an immortalized human bone marrow-derived MSC line (Y201)^37^ were used to assess the cytocompatibility of the hydrogels. The 3T3 cells were cultured in Dulbecco’s Modified Eagle’s Medium (DMEM) (Gibco), 1% penicillin–streptomycin (Sigma–Aldrich, P0781), and 10% fetal bovine serum (Sigma–Aldrich, F9665), while the MSCs were cultured in Minimum Essential Medium Eagle (αMEM) (Sigma-Aldrich, M4526), 10% fetal bovine serum (Sigma–Aldrich, F7524), 1% antibiotic/antimycotic solution (Sigma–Aldrich, A5955), 1% GlutaMAX (Life Technologies, 35050038), and 0.01% l-ascorbic acid 2-phosphate (Sigma–Aldrich, A8960). Cells were harvested when reaching 70–80% confluency. For cell counting, the cell suspension was mixed (1:1 ratio) with a 0.4% trypan blue solution (Bio-Rad) and cell counted using an automated cell counter (Cellometer Auto 1000, Nexcelom).

Hydrogel samples were removed from the fridge (4 °C) and prewarmed to 37 °C. Cells were encapsulated into the hydrogel by gentle pipetting to ensure an evenly mixed sample, at a final density of 1 × 10^6^ cells mL^–1^. 100 μL of cell-laden hydrogel was then dispensed into ThinCert inserts (Greiner Bio-One) in 24-well plates and cell culture medium (1 mL) was added into each well/insert. 250 μL was added to the top of the hydrogel surface in the inset, and 750 μL was added to the well. Cell culture plates were then incubated at 37 °C with 5% of the CO_2_. The growth medium was changed every 20 min during the first hour of the experiment and subsequently every 2 days.

### Live/Dead Assay

To assess cell viability in the hydrogels, the live/dead assay (Thermo Fisher Scientific) was used. The cell culture medium was removed, and the samples were washed 3 times with DPBS. A working solution of staining reagent was prepared in DPBS (4 μM ethidium homodimer I and 2 μM calcein-AM) and 100 μL was added to the top of the hydrogel surfaces (in the insets) and a further 600 μL into the wells. The cell culture plates were then incubated at 37 °C in 5% CO_2_ for 30 min before the staining reagent was removed and the samples were washed once more 1–3 times with DPBS. The cell-laden hydrogels were then transferred onto a microscope glass slide for imaging. Samples were imaged at days 0, 3, 7, and 14 by confocal laser scanning microscopy (Leica TCS SP8) with the following wavelength settings: green channel excitation/emission 494/517 nm and red channel excitation/emission 528/617 nm.

### PicoGreen Assay

To quantify the number of cells present in the hydrogels, the amount of cells’ dsDNA was quantified via PicoGreen assay (Invitrogen, Thermo Fisher Scientific). The cell culture medium was discarded, and the cell-laden hydrogels were transferred into a microcentrifuge tube. A 10× Pronase E (Sigma-Aldrich) solution (400 μL) was then added, and the sample was mixed using a vortex. The tube was placed in a 37 °C water bath, and the sample was agitated every 1–3 min until the gel was hydrolyzed evenly. Then, 500 μL of a 2× TE buffer containing 1% Triton X was added into the tube to lyse the cells. The tube was incubated at room temperature for 30 min, and the membrane was taken out before freezing the sample at −20 °C. When ready for analysis, the samples were removed from the −20 °C freezer, thawed at room temperature, and agitated until homogeneous. 100 μL samples were added into a black-walled 96-well plate (F-bottom, Greiner) with an equal volume of diluted PicoGreen reagent (200-fold in 1× TE buffer). As a background control, 1× TE buffer (100 μL) was mixed with an equivalent volume of the PicoGreen solution. The assay plate was incubated at room temperature in the dark for 5 min then measured using a plate reader (CLARIOstar), using fluorescence detection with excitation at 480–512 nm and emission at 520 nm. PicoGreen standard curves were prepared using the same experimental protocol as described earlier but using hydrogels in which known numbers of cells were encapsulated and left for 5–10 min before analysis. The experiments we performed in triplicate and the statistical analysis was performed in GraphPad Prism 8 using one-way nonparametric ANOVA and post hoc Tukey’s multiple comparisons tests to assess statistical significance via *P* values.

## Results and Discussion

**Figure 2:**
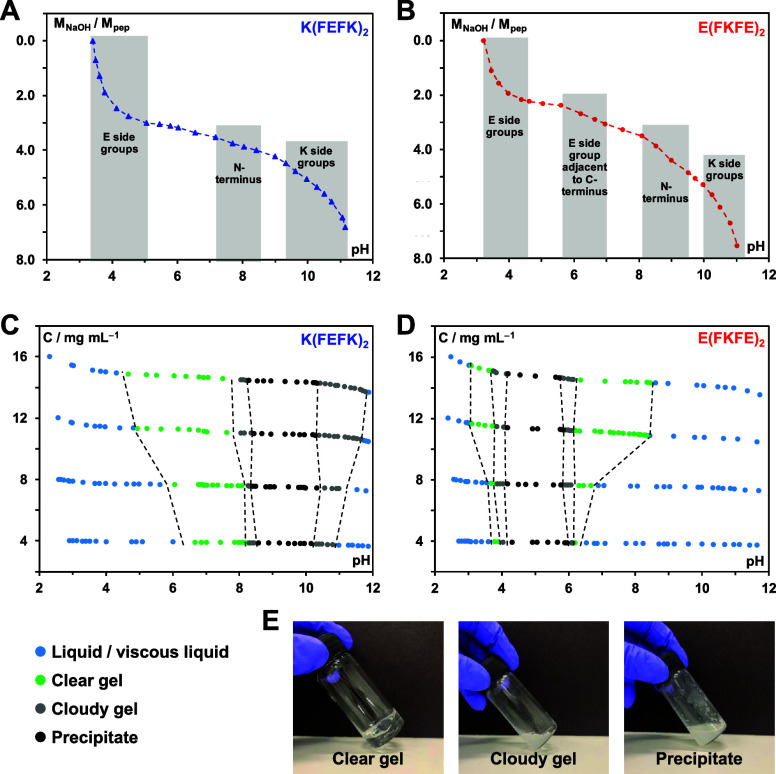
(A and B) Titration curves obtained at 2 mg mL^–1^ peptide concentration through stepwise addition NaOH. (C and D) Physical state vs concentration and pH phase diagrams obtained. (E) Photographs illustrating the appearance of the samples in the different identified physical states.

As stated in the introduction, these two peptides were designed to be “symmetrical” in relation to the position and distribution of their anionic (COOH/COO^–^) and cationic (NH_3_^+^/NH_2_) groups and to carry an overall theoretical net charge modulus of 1 at pH 7. As shown in our previous work, self-assembly can induce a shift in p*K*_a_ of these ionic groups depending on the surrounding environment created.^26,38^ The theoretical p*K*_a_ and the overall theoretical peptide charge vs pH curves are presented in Figure S1. To investigate the effect of self-assembly on p*K*_a_ of these groups, titration experiments were performed on each peptide at a 1 mg mL^–1^ concentration. The resulting curves are presented in Figure 2A, B. Both peptides were purchased as HCl salts; therefore, when dissolved in water low pH, ∼ 3.2, solutions were obtained. Upon addition of NaOH a first p*K*_a_-like transition was observed up to pH 5 for *K*(FEFK)_2_ and pH 4.5 for *E*(FKFE)_2_. This first transition is associated in both cases with deprotonation of the glutamic acid side groups. The terminal COOH groups are assumed to be already deprotonated as their theoretical p*K*_a_, 2.19 for *K*(FEFK)_2_ and 2.18 for *E*(FKFE)_2_, is significantly lower than the starting pH.

For *E*(FKFE)_2_, a second broad p*K*_a_-like transition is observed from pH 5.6 to 7. This transition is attributed to the “late” deprotonation of one of the carboxylic acid side chain groups, suggesting that not all three COOH groups have the same p*K*_a_ once self-assembly has occurred. Looking at the structure of *E*(FKFE)_2_, the COOH side chain group placed directly beside the terminal COO^–^ group will experience a very different environment compared with the two other groups, which are located between two amino groups. It is thought that the presence of the terminal COO^–^ creates a highly negative environment stabilizing the protonated form of the neighboring carboxylic acid side group leading to a higher apparent p*K*_a_. Two additional p*K*_a_-like transitions are observed above pH 7, the first one from pH 8.1 to 9.5 and the second one from pH 10.0 upward. These two transitions are assigned to the deprotonation of the NH_3_^+^ terminal group, suggesting a ∼1 unit shift compared with its theoretical p*K*_a_ of 9.97, and to the deprotonation of the lysine side chain amino groups, respectively, theoretical p*K*_a_ of 10.53.

For *K*(FEFK)_2_ after the first p*K*_a_-like transition, the next transition is observed from pH 7.2 to 8.5. For this peptide too, this transition is assigned to the deprotonation of the terminal NH_3_^+^ group, corresponding once again to a ∼1 p*K*_a_ unit shift compared with the theoretical p*K*_a_ which in this case is 8.95. For both peptides, it is hypothesized that when assembled in register into a cross β-sheet configuration (Figure S2), the terminal amine group is located in the proximity of the terminal phenylalanine of the next peptide and therefore in the proximity of the hydrophobic core of the β-sheet fiber. This hydrophobic environment is thought to destabilize in both peptides of the protonated form of the terminal amino group and lead to early deprotonation. Finally, at higher pH, the sharp p*K*_a_-like transition observed from pH 9.5 upward is assigned to the deprotonation of the lysine side chain amino groups.

To confirm the assignment discussed earlier, we built the physical-state phase diagram for each peptide as a function of concentration and pH (Figure 2C, D). As discussed in our previous work, for this family of peptides when the isoelectric point (pI) is reached, large-scale fiber aggregation leading to cloudy solutions and hydrogels and/or macroscopic phase separation is usually observed. For *K*(FEFK)_2_ based on the p*K*_a_ transition assignments made above when the pH increases from 3.2 to 7, the overall net charge carried by the peptide decreases from +3 (two glutamic acid side groups protonated, terminal carboxylic acid group deprotonated, all four amino groups protonated) to +1 (all three carboxylic acid groups deprotonated, all four amino groups protonated). As can be seen from Figure 2C, this change leads to the formation of clear solutions and hydrogels, depending on the concentration. From pH 7.2 upward as discussed earlier, the terminal amines are thought to protonate leading to a further decrease in net charge carried by the peptide from +1 to 0. This change coincides as expected with the formation of cloudy solution and hydrogels and macroscopic phase separation. Once the lysine amine side groups start to deprotonate above pH 10, the net charge of the peptide will go from 0 to −3 leading to the opposite sequence of physical-state changes, from macroscopic phase separation and cloudy hydrogels and solutions to clear hydrogels and solutions.

A similar correlation between peptide net charge, p*K*_a_ transitions, and physical state is observed for *E*(FKFE)_2_. In this case, when the pH increases, the peptide net charge goes from +2 at pH 3.2 (three glutamic acid side chain groups protonated, terminal carboxylic acid group deprotonated, all three amino groups protonated) to 0 at pH 5.0 (two side chain and terminal carboxylic acid groups deprotonated, one carboxylic acid side chain group, all four amino groups protonated). As can be seen from Figure 2D, this results in this case too, in a physical-state change from clear solutions and hydrogels to cloudy solutions and hydrogels, and eventually to macroscopic phase separation. When the pH increases above 5.0, the peptide net charge goes from 0 to −1 at pH 7 due to the deprotonation of the last carboxylic acid side chain group, then to −2 due to the deportation of the terminal amino group, and finally, to −4 above pH 10 due to deprotonation of the lysine amino side groups. As can be seen from Figure 2D, the reverse sequence of physical state changes is observed.

These results confirm the assignment made above for the p*K*_a_-like transitions observed and confirm the correlations between peptide charge and the sample's physical state. They also show that clear solutions and hydrogels are obtained at pH 7 for both peptides as predicted. For the remainder of this work, the samples' pH was always adjusted to 7.

**Figure 3:**
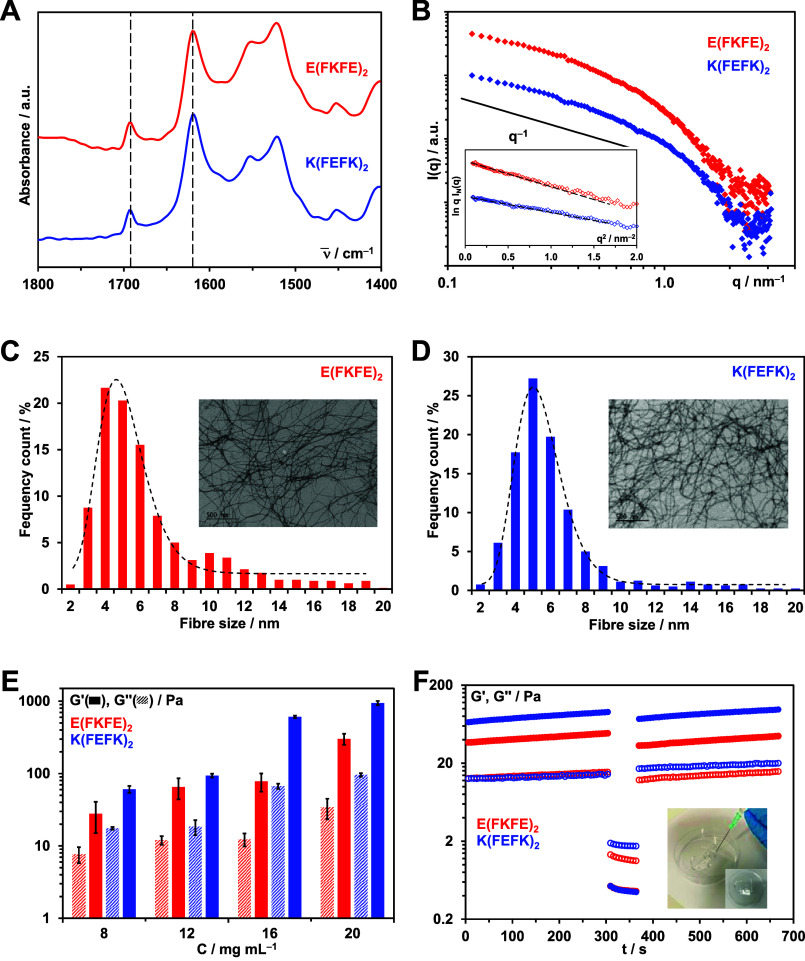
(A) ATR -FTIR spectra obtained for peptide hydrogels prepared at 12 mg mL^–1^; (B) SAXS patterns obtained for peptide hydrogels prepared at 6 mg mL^–1^. Insert: SAXS patterns presented in a ln qI(q) vs q^2^ Guinier representation and best fit (black dotted lines) obtained for the low q linear regions; (C and D) TEM images and fiber size distribution obtained (maximum size cutoff: 20 nm) for hydrogels prepared at 12 mg mL^–1^ and subsequently diluted 20 folds. Black lines represent the best log-normal fits of the size distributions obtained. (E) Storage, *G*′, and loss, *G*″, shear moduli (shear-strain: 0.2%, frequency: 1 Hz) vs peptide concentration plot. Original mechanical spectra are presented in Figure S3; (F) Storage, *G*′, and loss, *G*″, shear moduli (frequency: 1 Hz) of hydrogels prepared at 12 mg mL^–1^ vs time graph. Different shear stains were applied: 0.2% first 5 min; 1000% subsequent 1 min and 0.2% last 5 min. Insert: photographs of *E*(FKFE)_2_ hydrogel being injected through a 27G needle.

Next, we investigated the structural and mechanical properties of the hydrogels. The adoption by these peptides of β-sheet conformations was confirmed by ATR-FTIR. Indeed, as can be seen from Figure 3A, two absorption bands, characteristic of the adoption by proteins and peptides of β-sheet-rich conformations, a strong band at 1620 cm^–1^ and a weak band at 1694 cm^–1^, are clearly detected.

The formation of fibers and fibrillar entangle networks was confirmed by SAXS and TEM. As seen in Figure 3B for both peptides, the SAXS patterns show *q*^–1^ behavior at low *q*, typical of the scattering by fibers. From a Guinier representation, ln *qI(q)* vs *q*^2^, the fibers cross-section radius of gyration, *R*_σ_, can be obtained. Indeed, it has been shown that for thin rod-like structures for *qR*_σ_ < 1 the scattering intensity can be written as^34,39^
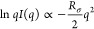
3

As can be seen from Figure 3B, insert linear behaviors are indeed observed at low *q* confirming that for both peptides the fibers formed can be considered as infinitely long thin rods. From the fitting of the linear region, *R*_σ_ values of 1.2 ± 0.2 nm for *K*(FEFK)_2_ and 1.4 ± 0.2 nm for *E*(FKFE)_2_ were obtained. Assuming a cylindrical geometry, *R*_σ_ can be related to the fiber diameter, *d*_f_, through:
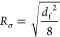
4

Estimated fiber diameters of 3.3 ± 0.5 nm for *K*(FEFK)_2_ and 4.0 ± 0.5 nm for *E*(FKFE)_2_ were obtained.

TEM images of diluted hydrogels confirmed the formation of entangled semiflexible fibrillar networks for both peptides (Figure 3C, D). A size analysis was performed to estimate the average diameter of the thinnest fibers (network basic fibers) observed. From the log-normal fits obtained distribution maxima of 4.9 ± 1.0 nm for *K*(FEFK)_2_ and 4.5 ± 1.6 nm for *E*(FKFE)_2_ were obtained in good agreement with SAXS results. The distributions of full-width at half-maximum (FWHM) were found to be 3.6 ± 0.6 and 3.2 ± 0.5 nm, respectively, suggesting relatively broad fiber widths distributions. These results suggest that some fiber thickening occurs in these systems probably though stacking via the hydrophilic faces.^18,40^

Large fiber aggregates can also be observed on the TEM images. The formation of larger structures does not seem to be supported by our SAXS data that suggest in the as-prepared samples the presence of fairly regular networks formed from thin fibers. The presence of these extended larger aggregated fiber bundles is typical of images obtained by TEM for this family of peptide hydrogels and is thought to be due to the sample preparation method used. Dilution and vortexing are used to reduce fiber density and access the basic fibers leading probably to the formation of these larger aggregated structures. They are still representative of the highly entangled nature of these networks.

Finally, the mechanical properties of the hydrogels were investigated. In Figure S3, the shear strain sweep curves obtained for both peptide hydrogels at different concentrations are presented. In the concentration range investigated mechanical spectra typical of gel-like materials were obtained for both peptides with *G*′ (storage) and *G*″ (loss) moduli being constant up to ∼1 to 10% shear strain depending on sample concentration and peptide used and *G*′ > *G*″ by ∼1 order of magnitude. At high shear stains, shear-thinning behavior is observed with the hydrogels “breaking” and eventually *G*″ > *G*′. In Figure 3E the shear moduli obtained at 0.2% strain are presented as a function of concentration. As expected for both peptides, the moduli increase with increasing concentration. A power low *G*′ ∝ *C*^2.3^ was obtained for *E*(FKFE)_2_ (Figure S4) in good agreement with Jones and Marques theory suggesting the formation of a self-consistent semiflexible network of thin fibers at all concentrations. For *K*(FEFK)_2_, a larger exponent is observed, *G*′ ∝ C^3.3^ (Figure S4) suggesting in this case a stronger tendency to form thicker fibers with increasing concentration.^22,24^

These materials show shear-thinning behavior at large shear strains. In order to investigate their injectability, tests were performed by using a 27G needle. The photographs in Figure 3F clearly show that these hydrogels can easily be injected through thin needles. To confirm that following injection the hydrogels recover their mechanical properties, the samples were submitted in situ, in the rheometer, to high shear stains, 1000% for 60 s, and the recovery of their mechanical properties followed. When the large shear strain is applied *G*″ > *G*′ suggesting liquid-like behavior. Upon the removal of the high shear strain, the hydrogels recover their original mechanical properties within a few seconds (Figure 3F) confirming the suitability of these materials for delivery through injection.

**Figure 4:**
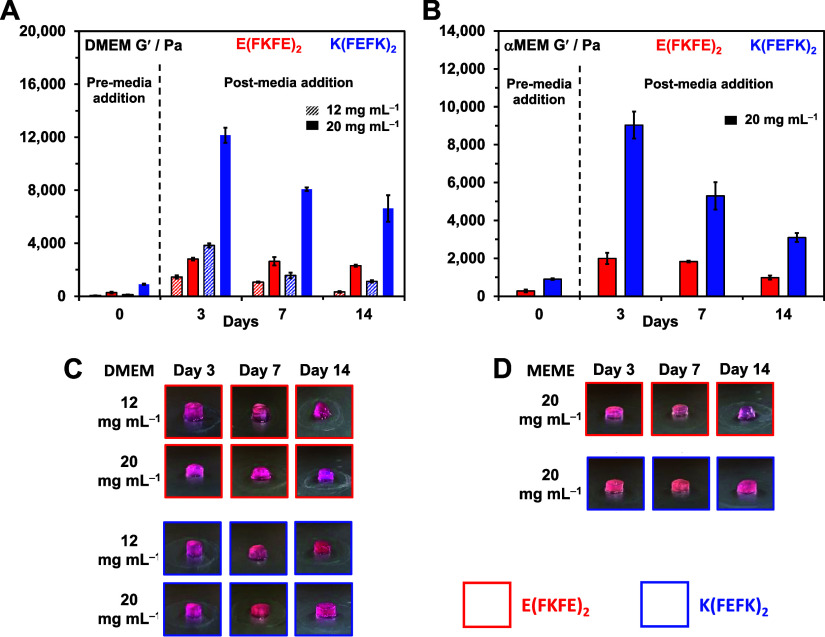
(A and B) Hydrogel shear storage moduli (*G*′) vs days of culture (no cells present) in the different cell culture media; (C and D) Photographs of hydrogels after extraction from cell culture inserts vs days of culture (no cells present) in the different cell culture media.

We investigated next the cytocompatibility of the hydrogels using two common cell lines, 3T3 mouse fibroblasts and human MSCs. It is well established that the addition of cell culture media to peptide-based hydrogels affects their mechanical properties. In our case, two different media were used DMEM for 3T3 fibroblasts and αMEM for MSCs. When tested in cell culture conditions without cells the hydrogels were found to be significantly stiffer at day 3, following the addition of the media (Figure 4A, B). As discussed in our previous work the increase in hydrogel moduli is due to the presence in the media of salts that promote charge screening effects and fiber bundling leading to increased cross-linking density.^20,41,42^ Stiffening was found to be more marked in DMEM and for *K*(FEFK)_2_ hydrogels reflecting differences in media composition and peptide fiber surface charge, respectively. The hydrogel moduli were found to decrease to various degrees over time depending on the peptide and concentration used. The weakening of the hydrogels is thought to be due to a combination of hydrogel swelling, peptide dissolution, and physical erosion resulting from the frequent media changes. The *E*(FKFE)_2_ hydrogels after 14 days show some loss of integrity and loss of shape when extracted from the cell culture inserts, while *K*(FEFK)_2_ hydrogels keep their integrity and shape (Figure 4C, D). These results point toward a lower stability of the negative hydrogels in cell culture media.

**Figure 5:**
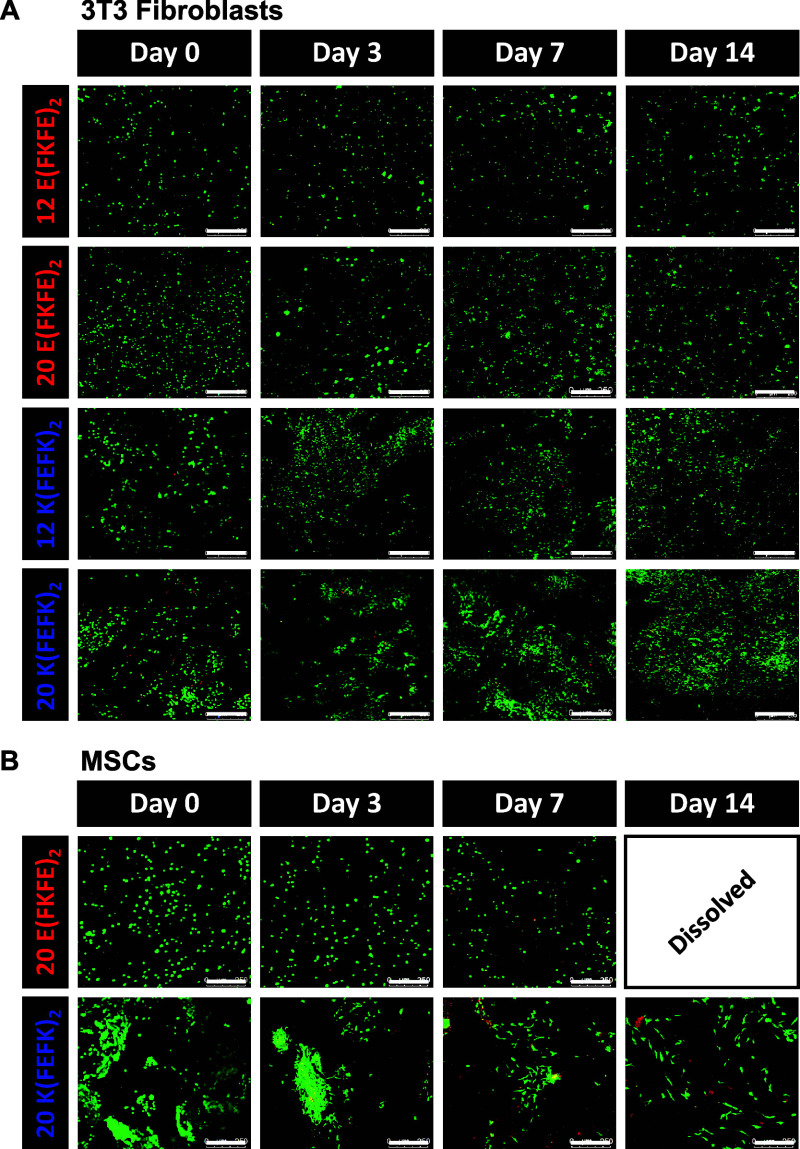
Live/dead images obtained for (A) 3T3 fibroblasts and (B) MSCs cultured for 14 days in the hydrogels (3D culture). Scale bar: 250 μm.

**Figure 6:**
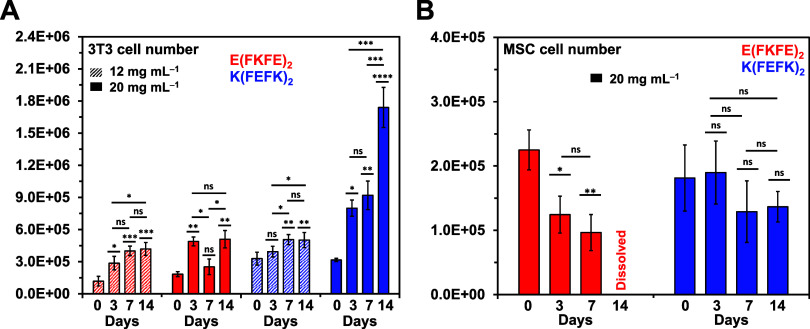
Cell number vs days of 3D culture in hydrogels obtained via PicoGreen DNA assay for (A) 3T3 cells and (B) MSCs. The results presented as mean ± standard deviation. Statistical significance was assessed via *P* values: *P < 0.05, **P < 0.01, ***P < 0.001, ****P < 0.0001, and “ns” no significant differences. Statistical data presented just above bars were measured in relation to day 0.

For both peptides, cells were encapsulated in the hydrogels through gentle mixing, and 3D cell culture was performed over 14 days. Good viability was observed for 3T3 fibroblasts in all hydrogels (Figure 5A) across the 14 days of culture. Significant proliferation was observed only for the *K*(FEFK)_2_ hydrogel prepared at 20 mg mL^–1^ (Figure 6A). It is well established that positively charged and stiffer scaffolds (>5 kPa) promote fibroblast proliferation.^43^ For the other significantly weaker hydrogels (<3 kPa), cell numbers were observed to increase from days 0 to 3 and then to become stable.

As far as MSCs were concerned, they were found to be difficult to disperse in the positive hydrogel *K*(FEFK)_2_ and formed cell clumps following encapsulation (Figure 5B). Viability was found to be good, although no significant change in cell number was observed (Figure 6B) over 14 days. A marked change in cell morphology was seen at days 7 and 14 with the cells becoming elongated and spindle-like. In the negative hydrogel *E*(FKFE)_2_, MSCs viability was observed to be higher with less dead cells being observed at days 3 and 7 (Figure 5B) although after 14 days the hydrogels were found to dissolve. A decrease in cell number was observed over the 14 days (Figure 6B) which is thought to be linked to the degradation of the material. These results point toward MSCs actively degrading the negative scaffold.^44^

Overall, these preliminary experiments show that these hydrogels are cytocompatible and suitable for a 3D cell culture. They also highlight the importance of scaffolds’ physicochemical properties, such as modulus and fiber charge, when engineering cell-scaffold culture systems.

Next, we incorporated in the hydrogels two polymers, poly-l-lysine (28kPLys) and dextran (40kDex), which carry positive and negative charges at neutral pH, respectively (Figure 1B, C and Table 1). First, we investigated whether the introduction of polymers affected the morphology of the hydrogels. As can be seen from Figure S5 in this case too for all four polymer-loaded hydrogels, a strong absorption band at 1620 cm^–1^ and a weaker absorption band at 1694 cm^–1^ were observed in the FTIR spectra, confirming that the incorporation of the polymers did not affect the adoption by the peptides of β-sheet conformations.

**Figure 7:**
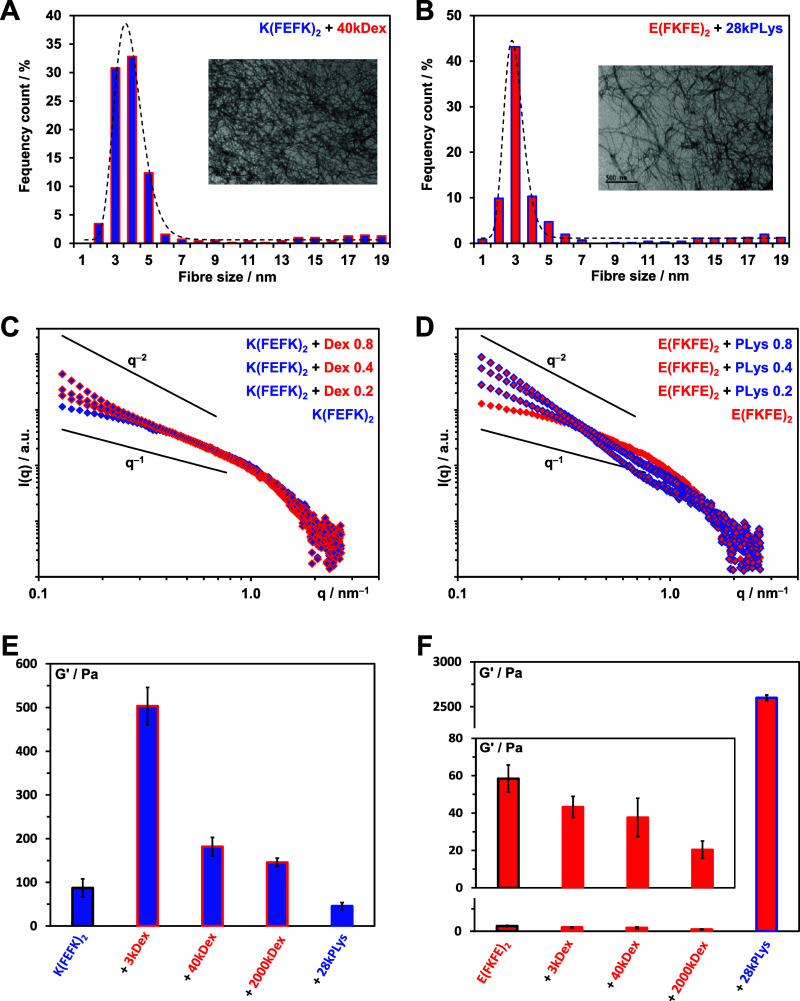
(A and B) TEM images and corresponding fiber width distributions (maximum size cutoff: 20 nm) obtained for polymer-loaded hydrogels prepared at 12 mg mL^–1^ peptide and 0.8 mg mL^–1^ polymer concentrations. Black line represented the best log-normal fits of the fiber width distributions obtained. (C and D) SAXS patterns obtained for polymer-loaded hydrogels prepared at 6 mg mL^–1^ peptide and varying polymer concentrations. (E and F) Storage, *G*′, and shear moduli (shear-strain: 0.2%, frequency: 1 Hz) of the peptide and polymer-loaded hydrogels prepared at 12 mg mL^–1^ peptide and 0.8 mg mL^–1^ polymer concentrations.

The formation of fibers was confirmed by TEM. In all cases, entangled networks of semiflexible fibers were observed (insets Figures 7A, B and S6). The fiber width distributions are presented in Figures 7A, B and S6 with the best log-normal fits obtained. The distributions maxima and FWHM for all 6 samples, including pure peptide hydrogels (Figure 3C, D), are listed in Table 2. When the polymers and the peptide fibers carry similar charges, i.e., *E*(FKFE)_2_ + 40kDex and *K*(FEFK)_2_ + 28kPLys, no significant changes in distribution maxima and FWHM’s are observed suggesting that the addition of the polymers did not affect the peptide fibers morphology. The polymers are thought to be simply dissolved in the liquid phase of the hydrogels. This was confirmed by SAXS; as can be seen from Figure S7, no changes in scattering patterns were observed suggesting that the addition of the polymers did not affect the network topology formed, pointing toward the absence of interactions between peptide fibers and polymers.

**Table 2:** Comparison of Fiber Width Distributions Maxima and Full-Width at Half-Maximum

sample name	nominal charge	fiber widths distribution maximum (nm)	fiber widths distribution full-width at half-maximum (nm)
*E*(FKFE)_2_	(−)	4.5 ± 0.9	3.6 ± 0.6
*E*(FKFE)_2_ + 40kDex	(− −)	3.6 ± 0.7	3.1 ± 0.4
*K*(FEFK)_2_ + 40kDex	(+ −)	3.5 ± 0.7	1.9 ± 0.3
*K*(FEFK)_2_	(+)	4.9 ± 1.0	3.2 ± 0.5
*E*(FKFE)_2_ + 28kPLys	(− + )	2.8 ± 0.4	1.4 ± 0.3
*K*(FEFK)_2_ + 28kPLys	(+ + )	3.2 ± 0.6	3.7 ± 0.5

For the polymer-loaded hydrogels in which the peptide fibers and polymers carry opposite charges, i.e., *E*(FKFE)_2_ + 28kPLys and *K*(FEFK)_2_ + 40kDex, a significant reduction in FWHM was observed suggesting that the polymers prevent the formation of overall thicker basic fibers. This points to the presence of strong electrostatic interactions. It is thought that during β-sheet fiber formation (two cross β-sheet strands coming together through their hydrophobic faces), the oppositely charged polymers interact with the charged hydrophilic fiber surfaces resulting in the formation of a peptide fiber–polymer complex where the polymer wraps around the β-sheet fiber. The polymer is thought to then prevent the thickening of the fibers through their hydrophilic surfaces.^45^

The SAXS patterns obtained for these two samples clearly show that the introduction of an oppositely charged polymer in the hydrogels also has a strong effect on the hydrogels network topology. Indeed, for *K*(FEFK)_2_ + 40kDex hydrogels, an increase in low-*q* scattered intensity with increasing polymer content is observed, while for *E*(FKFE)_2_ + 28kPlys, in addition to the increase in low-*q* scattering, an overall shift of the scattering pattern toward lower *q* values is observed. For both of these systems, these changes in scattering patterns are consistent with the formation of larger structures, such as fiber aggregates and bundles. This is supported by the change in appearance of the hydrogel, which became more turbid with increasing polymer content. When wrapping around the peptide fibrils as suggested earlier, the polymer is thought to screen the surface charges, and therefore, the overall peptide fiber–polymer complex is expected to carry a significantly lower surface charge leading to an increase in fiber aggregation and large bundle formation via hydrophobic interactions.

The differences in the level of interactions and therefore levels of structural changes observed via SAXS for 28kPLys and 40kDex systems are thought to reflect the differences in overall charge levels and charge distributions of the two polymers. For 28kPLys, the positive charges arise from the presence of a lysine amino side group on each monomer along the polymer chain. This results in a homogeneous positive charge distribution along the polymer chain, leading to strong interactions and high levels of charge screening. For 40kDex, the negative charge is imparted by the FITC functionalization. The level of functionalization as given by the manufacturer (0.024–0.008 per monomers) suggests that significant polymer chain segments remain unfunctionalized and therefore uncharged resulting in overall weaker interactions with the peptide fibers and reduced charge screening effects.

The differences in peptide fibers–polymers interactions were also reflected in the mechanical properties of the polymer-loaded hydrogels. As discussed in our previous studies, the formation of fiber aggregates and bundles will result in an increase in network cross-linking level and cross-links stability and therefore is usually associated with an increase in mechanical properties.^24,25^ For both the *K*(FEFK)_2_ + 40kDex and *E*(FKFE)_2_ + 28kPLys hydrogels, a significant increase in *G*′ was indeed observed compared to the pure peptide hydrogels (Figure 7E, F). This increase was particularly marked for the *E*(FKFE)_2_ + 28kPLys system, confirming the presence of stronger interactions and charge screening effects in this system.

For the dextran-loaded *K*(FEFK)_2_ hydrogels, the increase in mechanical properties was found to be a function of the polymer molecular weight, with lower *M*_w_ dextran leading to larger increases in *G*′. This is thought to reflect the significantly higher FITC functionalization required for the lower *M*_w_ dextran (Table 1) as well as its higher mobility and reduced entrapment through entanglements. These will lead to stronger interactions and charge screening effects resulting in increased fiber aggregation and bundling and higher *G*′ for lower *M*_w_ dextran.

When a polymer with the same charge as the peptide fibers is added to the hydrogels, a small reduction in mechanical properties is observed reflecting the presence in the water phase of the charged polymers. This will lead to an increase in overall electrostatic repulsion across the system resulting in a slight decrease in cross-linking levels and stability. For the dextran polymers, the effect was found in this case too to be M_W_-dependent with a slightly more marked decrease in *G*′ being observed for the higher M_W_ dextran reflecting the effect of the polymer's overall hydrodynamic sizes.

**Figure 8:**
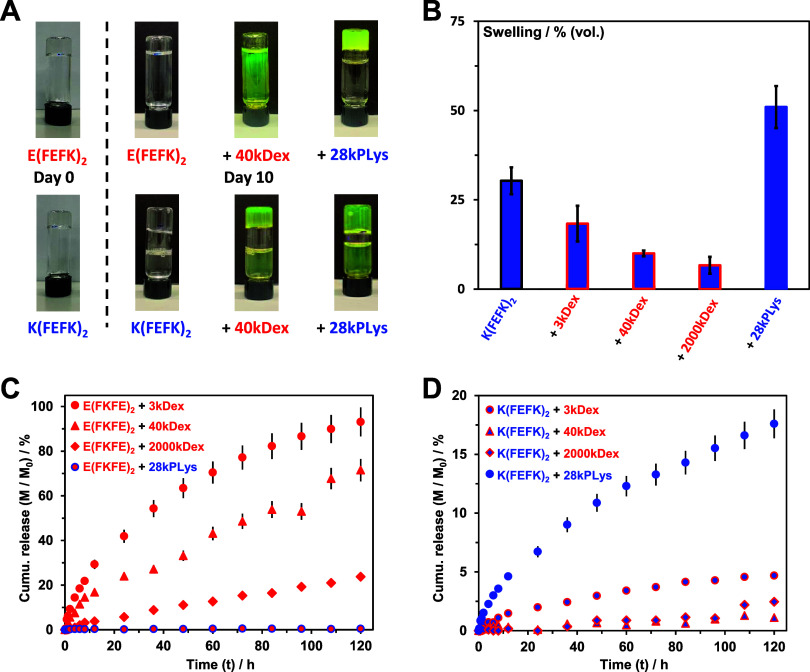
(A) Photograph of inverted vials at day 0 and after 10 days; (B) swelling ratio of K(FEFK)_2_ peptide and polymer-loaded hydrogels; and (C and D) polymers cumulative release curves vs time. All hydrogels were prepared at 12 mg mL^–1^ peptide and 0.8 mg mL^–1^ polymer concentrations.

Next, we investigated whether the addition of the polymers affected the long-term stability of the hydrogels. For this purpose, the hydrogels were placed in glass vials, and water was added on top in a 1:3 volume ratio. The stability of the hydrogels was checked after 10 days. As can be seen from Figure 8A, the *E*(FKFE)_2_ hydrogel was unstable and after 10 days dissolved. The addition of dextran (the same charge) did not change the outcome. On the other hand, the addition of 28kPLys stabilized the hydrogel, and after 10 days, no dissolution nor swelling was observed. As discussed earlier, the introduction of the oppositely charged polymer results in the formation of more and stronger cross-links preventing swelling.^24,25^

*K*(FEFK)_2_ hydrogel was found to be more stable with ∼30% (v/v) swelling being observed after 10 days. When 28kPLys was added (same charge), an increase in swelling at day 10 was seen up to ∼50%. On the other hand, when dextran (opposite charge) was added a reduction in swelling was observed with the higher *M*_w_ dextran leading to the largest reduction, down to ∼5% (v/v) (Figure 8B). The higher *M*_w_ dextran is expected to be able to cross-link a higher number of peptide fibers through electrostatic interaction and entanglements resulting in an increased network resistance to swelling.

These results show that the addition of small amounts of oppositely charged polymers can be used not only to increase the mechanical properties of peptide hydrogels but also to improve their long-term stability.

Finally, using the same experimental setup as earlier, we investigated the diffusion of the polymers from the hydrogels into the 1 mL top layer of the supernatant over 5 days using UV absorbance spectroscopy. Although some swelling in the *E*(FKFE)_2_ hydrogels and their dextran-loaded versions was observed, a clear supernatant liquid phase was still present. In Figure 8C, D, the release curves for all 8 polymer-loaded hydrogels are presented. As expected, the diffusion of the polymers from the hydrogels into the supernatants is dependent on the nature of the interactions present between peptide fibers and polymers as well as the polymers’ hydrodynamic radii and therefore their *M*_w_.^46,47^

To extract quantitative data from the release curves and minimize the effect of swelling, the cumulative fraction of polymer released over the first 24 h was plotted as a function of *t*^1/2^ (Figure S8). As for all sample linear behaviors were observed, we decided to use the non-Fickian diffusion model first proposed by Higuchi to analyze our data:
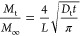
5where *M*_*∞*_ and *M*_t_ are the moles of polymer loaded into the hydrogels and released at time *t*, respectively, *L* is the thickness of the sample, in our case 9 mm, and *D*_t_ is the diffusion coefficient in m^2^ s^–1^. Although the model was originally developed by Higuchi to describe the dissolution and diffusion of a drug out of a matrix,^48^ it was subsequently shown by Rigter and Peppas to also apply to the diffusion of soluble drugs out of hydrogel slabs.^49,50^ One of the assumptions in this model is that the drug is significantly smaller than the mesh size of the matrix. For the 2000kDex as a linear behavior was still observed, we decided to use the model to extract an apparent diffusion coefficient that will include in this case the effect of polymer entrapment in the peptide fiber network. As part of the fitting procedure, a delay time was added to eq 5 reflecting the time required for diffusion of the polymers out of the hydrogel to start. The fitting parameters obtained are summarized in Table 3.

**Table 3:** Fitting Parameter: Diffusion Coefficient, Delay Time, and *R*^2^, Corresponding to the Best Linear Fits Obtained (Figure S8) Using Eq 5

probe	diffusion coefficient *D*_t_ (m^2^ s^–1^)	delay time (s)	*R*^2^
*E*(FKFE)_2_			
3kDex	(3.9 ± 1.3) × 10^–7^	700 ± 50	0.99
40kDex	(1.3 ± 0.7) × 10^–7^	737 ± 40	0.99
2000kDex	(6.8 ± 2.2) × 10^–9^	1170 ± 90	0.94
28kPLys	∼ 0^a^		
*K*(FEFK)_2_			
3kDex	(8.5 ± 1.9) × 0^–10^	480 ± 30	0.98
40kDex	∼ 0^a^		
2000kDex	∼ 0^a^		
28kPLys	(9.7 ± 2.2) × 10^–9^	555 ± 30	0.99

a No statistical meaningful release of polymers was observed during the first 24 h.

When loaded into the negative hydrogel *E*(FKFE)_2_, 28kPLys was found to become trapped with no statistically significant release over 5 days. On the other hand, when loaded into the positive hydrogel *K*(FEFK)_2_ 28kPLys was found to steadily diffuse out of the hydrogel with 18% (mol.) of the polymer being released after 5 days.

A similar overall diffusion behavior was observed for the dextran. 40kDex and 2000kDex were essentially trapped in the positive hydrogel *K*(FEFK)_2_ with minimal release detected after 5 days, while a low level of diffusion was observed for the 3kDex with 5% being released after 5 days. As discussed earlier, the difference in residual release observed between 28kPLys and dextran when loaded in *E*(FKFE)_2_ and *K*(FEFK)_2_, respectively, after 5 days is thought to be due to the lower overall number of charges carried by the dextran polymer chains and, therefore, lower levels of interactions with the oppositely charged peptide fiber network. It should also be noted that the number of charges per dextran polymer chain decreases statistically with decreasing *M*_w_ (Table 1), and therefore, a higher residual release at day 5 is observed for the lower *M*_w_ dextran.

When loaded in the negative hydrogel, *E*(FKFE)_2_ dextran was found to diffuse out readily. The amount of dextran release was found to be a function of the polymer *M*_w_ as larger molecules diffuse at slower rates out of the hydrogel network. In the case of the 2000kDex, some level of physical entrapment and entanglement cannot be excluded as the hydrodynamic size of the polymer is of the same order of magnitude as the hydrogel network mesh size.

Finally, the significantly higher diffusion rate obtained for the 40kDex out of *E*(FKFE)_2_ compared to found between 28kPLys out of *K*(FEFK)_2_ is thought to be related to the lower stability of the network junctions as demonstrated by the resulting swelling discussed earlier. Swelling will result in an increase in the mesh size and, therefore, in higher diffusion rates.

## Conclusions

We have designed based on the knowledge derived from our previous work two new peptide sequences, *E*(FKFE)_2_ and *K*(FEFK)_2_, which form stable hydrogels at pH 7 with oppositely charged fibers and networks. We demonstrated once more the direct link between charges carried by the peptides, media pH, and sample physical state. When the charge modulus carried by the peptides is decreased above one, significant peptide fiber aggregation and peptide fiber bundle formation are observed leading to the formation of cloudy hydrogels and precipitates. On the other hand, when the charge carried by the peptide is increased above one, clear hydrogels form above the critical gelation concentration, which is found to increase with increased peptide charge. We then investigated the cytocompatibility of the hydrogels and their suitability for 3D cell culture by culturing 3T3 mouse fibroblasts and bone marrow-derived human mesenchyme stem cells over 14 days. 3T3 was found to be viable in all hydrogels but to proliferate significantly only in the stiffer positive *K*(FEFK)_2_ hydrogel. MSCs were found to have good viability in both hydrogels, too, but were found to disperse readily in the negative *E*(FKFE)_2_ hydrogel while forming cell clumps in the positive *K*(FEFK)_2_ hydrogel. In the latter, a change in cell morphology was also observed from round to elongated. We then introduced charged polymers in the hydrogels’ formulations. When adding oppositely charged polymers strong electrostatic interactions lead to the formation of peptide fiber-polymer complexes resulting in thinner elemental fibers that aggregate and form bundles more readily leading to the formation of hydrogels with higher shear moduli. We finally showed that the diffusion of the polymers out of the hydrogel was directly linked to the nature of the electrostatic interactions existing between the peptide fiber networks and the polymers.

These results clearly show how the balance between peptide fibers' electrostatic repulsion and hydrophobic attraction needs to be controlled and tuned to design stable transparent peptide hydrogels. They also highlight the need to tailor not only the mechanical properties, which have been the focus of significant work in the literature but also the hydrogel physicochemical properties, such as fibers and networks charge, to the cell cultured. This present work also demonstrates how charged polymers can be used to tailor the properties of peptide hydrogels through guided intermolecular interactions and in particular increase their mechanical properties as well as their stability and resistance to swelling. These are key parameters to be controlled to design suitable 3D cell culture scaffolds and for the design of injectable hydrogel delivery systems that allow control of drug and cell release over time. This work offers new avenues for the design of self-assembling peptide hydrogels and demonstrates their potential for use in the biomedical field.

## Supporting Information Available

Theoretical overall charge carried by a peptide vs pH, Schematic representation of the peptides assembled in a register into cross β-sheet configurations, shear strain amplitude sweep curves, log–log plot of hydrogels storage moduli (*G*′) vs concentration, ATR-FTIR spectra obtained for polymer-loaded hydrogels, TEM images and fiber width distributions obtained for polymer-loaded hydrogels, SAXS patterns obtained for polymer-loaded hydrogels, and polymers cumulative release curve vs square root of time and 40kDex cumulative release curves vs time obtained at higher peptide concentrations (PDF)
