# Supplementary material for: Effect of Peptide–Polymer Host–Guest Electrostatic Interactions on Self-Assembling Peptide Hydrogels Structural and Mechanical Properties and Polymer Diffusivity
Source: Biomacromolecules. 2024 May 21;25(6):3628–41. doi: 10.1021/acs.biomac.4c00232 (PMC11170954; doi:10.1021/acs.biomac.4c00232)
Supplement: Supplementary file 1 — bm4c00232_si_001.pdf [file bm4c00232_si_001.pdf]

# Effect of peptide-polymer host-guest electrostatic interactions on self-assembling peptide hydrogels structural and mechanical properties and polymer diffusivity.

Siyuan Dong[1,2], Sam L. Chapman[3], Alain Pluen[3], Stephen M. Richardson[4], Aline F. Miller[1,2], Alberto Saiani[2,3*]

## Supporting Information

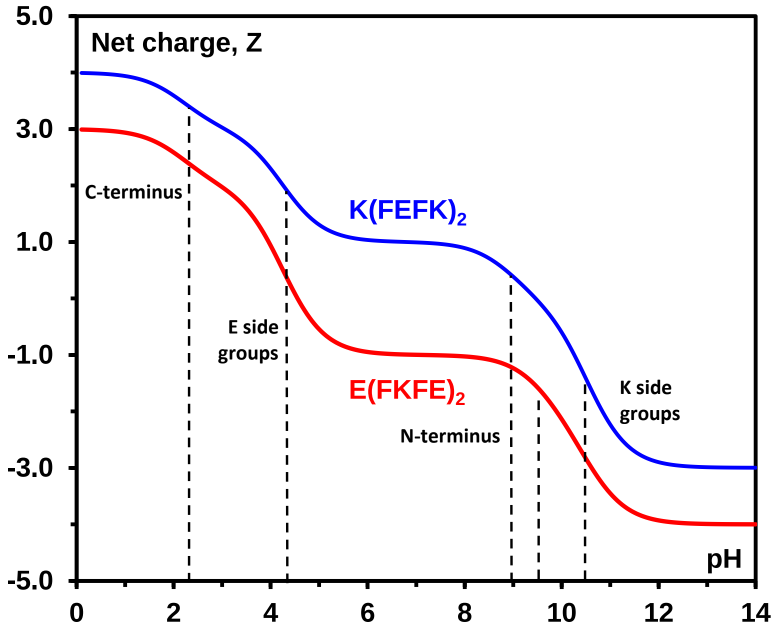


**Figure SI 1:** The overall charge carried by a peptide was calculated using the following equation:

$$Z = \sum_i N_i \frac{10^{pKa_i}}{10^{pH}+10^{pKa_i}} - \sum_j N_j \frac{10^{pH}}{10^{pH}+10^{pKa_j}} \qquad (1)$$

where $N_{i/j}$ are the numbers and $pK_{a\ i/j}$ the $pK_a$ values of the basic (i – $pK_a$ > 7) and acidic (j – $pK_a$ < 7) groups present respectively. The ionic groups present on the peptides are carboxylic acid (COOH / $COO^-$) at the C-terminus (theoretical $pK_a$ 2.18 and 2.19 on K and E side, respectively) and on the glutamic acid side chains (theoretical $pK_a$ 4.25), and amine ($NH_3^+$ / $NH_2$) at the N-terminus (theoretical $pK_a$ 8.95 and 9.97 on K and E side, respectively) and on the lysine side chains (theoretical $pK_a$ 10.53).

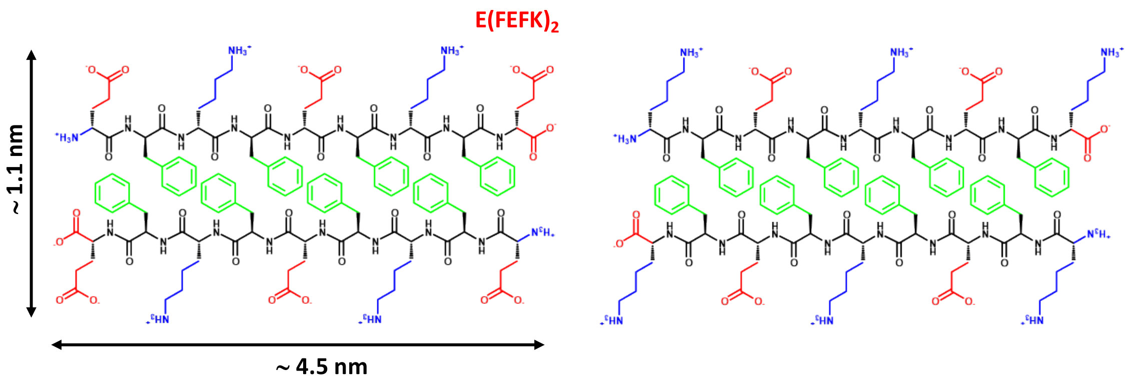


**Figure SI 2:** Schematic representation of the peptides assembled in register into cross β-sheet configurations

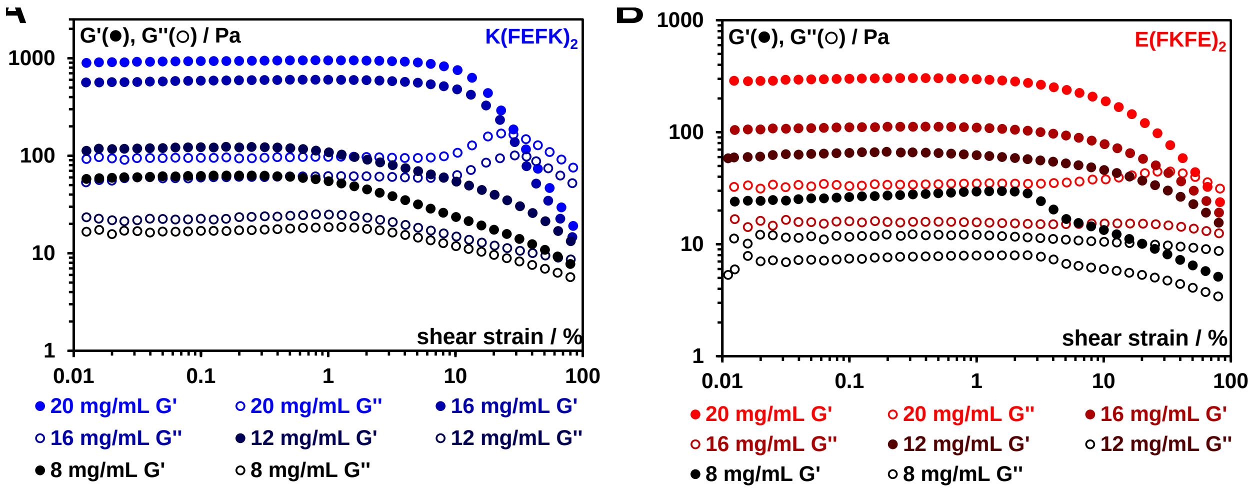


**Figure SI 3:** Shear strain amplitude sweep curves obtained at 1 Hz and 25°C for K(FEFK)$_2$ (left) and E(FKFE)$_2$ (right) hydrogels at 8, 12, 16 and 20 mg mL$^{-1}$ concentration.

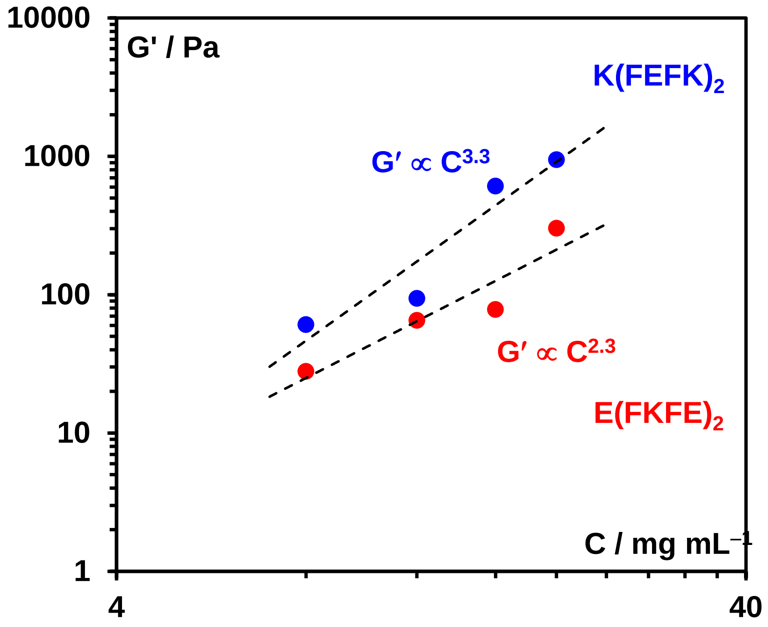


**Figure SI 4:** Log-log plot of hydrogels storage moduli (G′) vs concentration. Dashed line represent the best fits obtained.

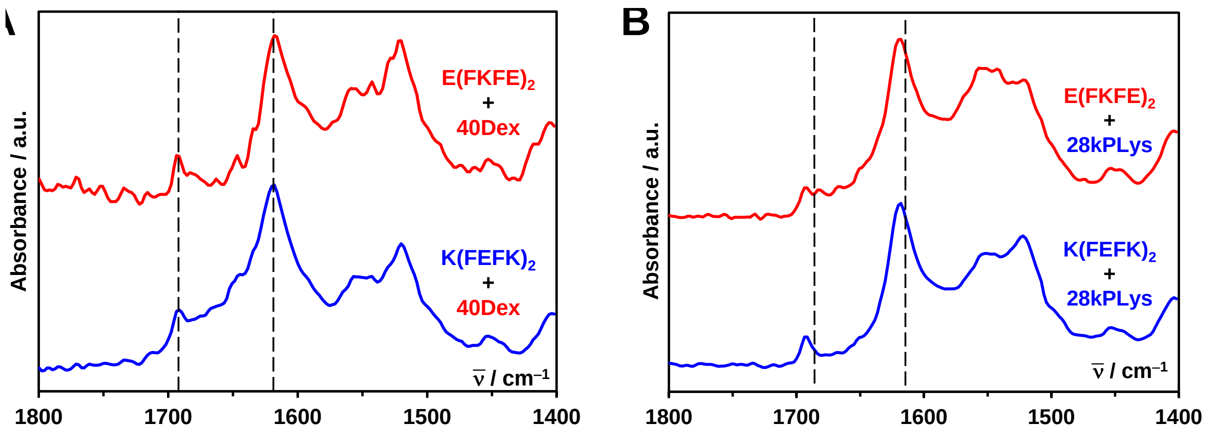


**Figure SI 5: A & B)** ATR -FTIR spectra obtained for peptide hydrogels prepared at 12 mg mL$^{-1}$ peptide concentration and 0.8 mg mL$^{-1}$ polymer concentration.

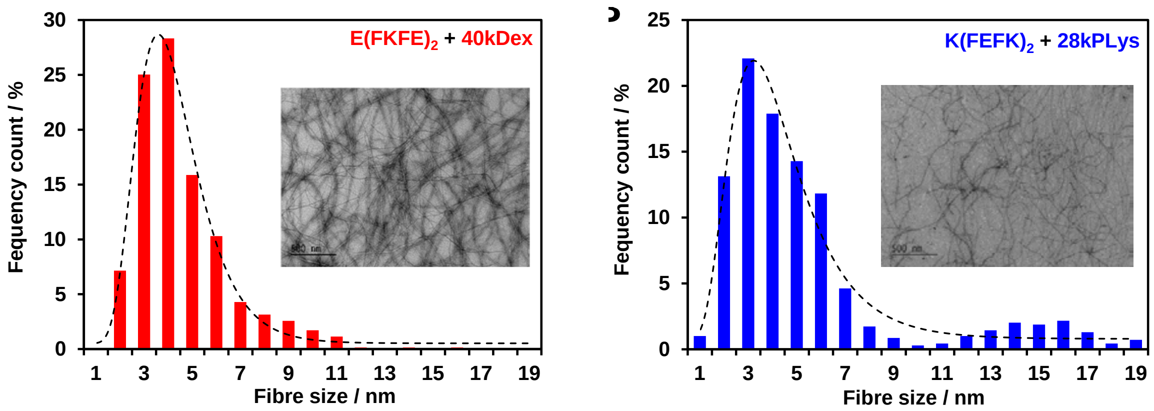


**Figure SI 6: A & B)** TEM images and fiber width distributions obtained (maximum size cut-off: 20 nm) for polymer-loaded hydrogels prepared at 12 mg mL$^{-1}$ peptide and 0.8 mg mL$^{-1}$ polymer concentration. Black line represented the best lognormal fits of the size distributions obtained.

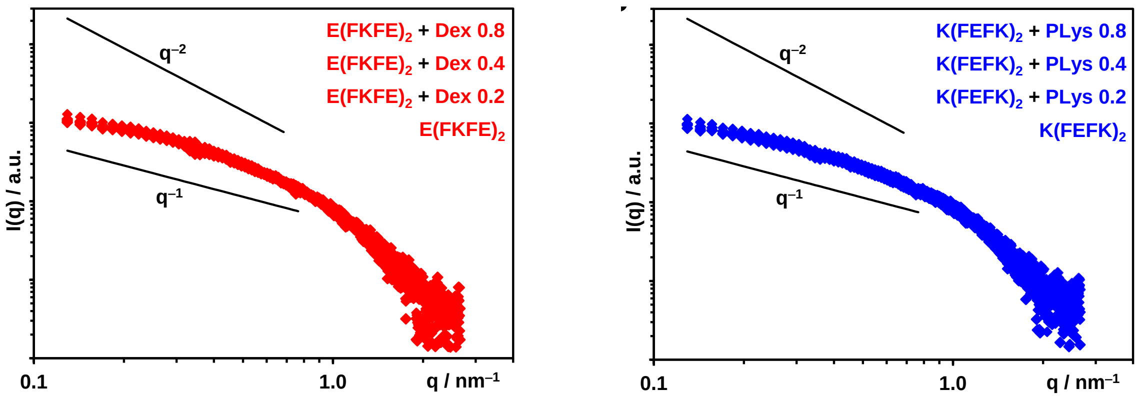


**Figure SI 7: A & B)** SAXS patterns obtained for polymer-loaded hydrogels prepared at 6 mg mL$^{-1}$ peptide and varying polymer concentrations.

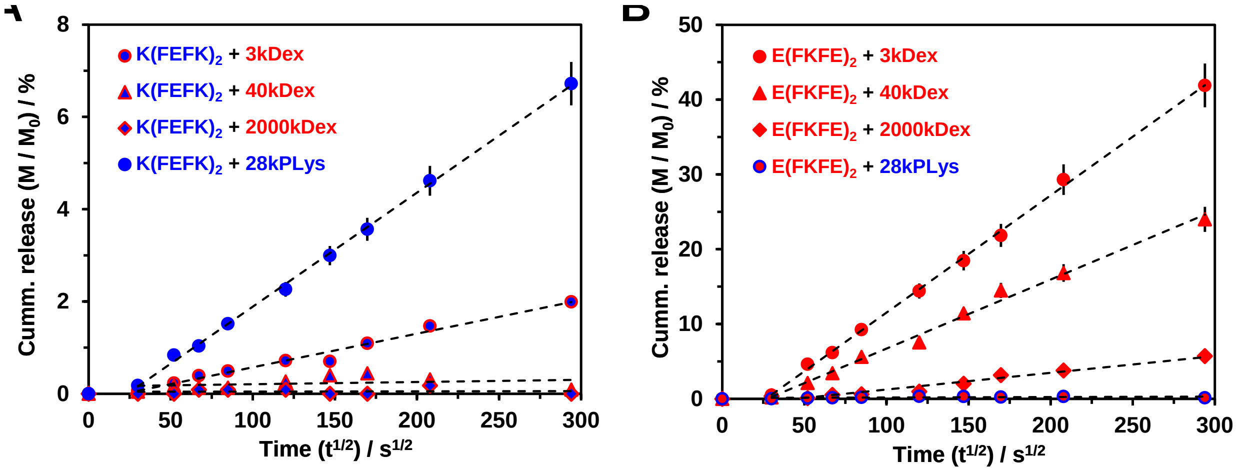


**Figure SI 8: A & B)** Polymers cumulative release curve vs square root of time ($t^{0.5}$). Dashed lines represent the best linear fits obtained.

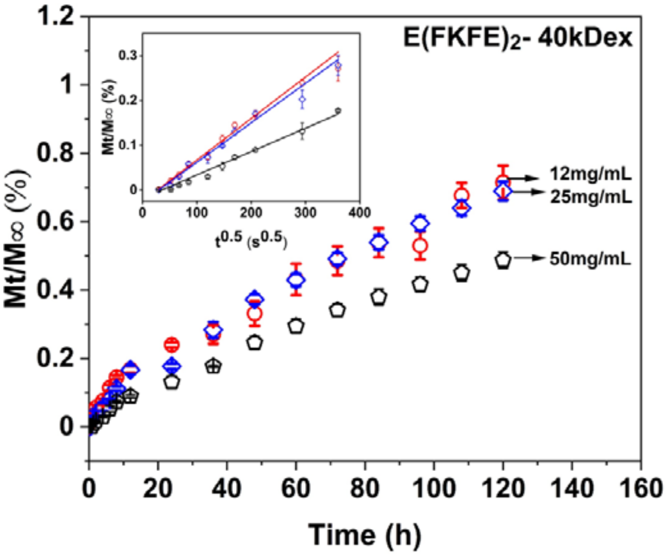


**Figure SI 9:** 40kDex cumulative release curves vs time. hydrogels were prepared at varying peptide and 0.8 mg mL$^{-1}$ polymer concentrations**;** Insert: 40kDex cumulative release curve vs square root of time ($t^{0.5}$). Dashed lines represent the best linear fits obtained.
